# Effects of exposure to nanoparticle-rich diesel exhaust on 8-OHdG synthesis in the mouse asthmatic lung

**DOI:** 10.3892/etm.2013.1198

**Published:** 2013-07-02

**Authors:** MICHITAKA TANAKA, HIROHISA TAKANO, YUJI FUJITANI, SEISHIRO HIRANO, TAKAMICHI ICHINOSE, AKINORI SHIMADA, KEN-ICHIRO INOUE

**Affiliations:** 1 Center for Medical Science, International University of Health and Welfare, Ohtawara, Tochigi 324-8501, Japan; 2 Department of Environmental Engineering, Graduate School of Engineering, Kyoto University, Nishikyo-ku, Kyoto 615-8540, Japan; 3 Research Center for Environmental Risk, National Institute for Environmental Studies, Tsukuba, Ibaraki 305-8506, Japan; 4 Department of Health Science, Oita University of Nursing and Health Science, Oita 871-1201, Japan; 5 Department of Veterinary Pathology, Tottori University, Tottori 680-8553, Japan

**Keywords:** nanoparticle-rich diesel exhaust, allergic pulmonary inflammation, oxidative stress, 8-OHdG

## Abstract

It has been demonstrated that exposure to diesel exhaust (DE) is associated with the induction and exacerbation of respiratory disorders; however, the impacts of DE containing mainly nanoparticles have been less studied. We have previously demonstrated that inhalation exposure to nanoparticle-rich DE (NR-DE) exacerbated allergic pulmonary inflammation, in the context of enhanced local expression of proinflammatory molecules. However, the underlying mechanisms have not been fully elucidated. 8-Hydroxydeoxyguanosine (8-OHdG) is a marker of oxidative damage, particularly in DNA. This study examined the effects of NR-DE on 8-OHdG synthesis in the lung in the presence or absence of an allergen. Institute for Cancer Research (ICR) mice were exposed by inhalation to four different gas compositions (control air, low-concentration DE, high-concentration DE and high-concentration DE without particulate matter) for 8 weeks, in the presence or absence of repetitive intratracheal administration of ovalbumin (OVA). Thereafter, we assessed the levels of 8-OHdG synthesis and expression in the lungs by means of enzyme immunoassay (EIA) and immunohistochemistry. The EIA revealed that the level of 8-OHdG was significantly higher in the high-concentration NR-DE-exposed and allergen-sensitized/stimulated group compared with that in the control air-exposed and allergen-treated group. The immunohistochemistry results demonstrated that the level of immunoreactive 8-OHdG was higher in the NR-DE-exposed and allergen-treated lungs compared with that in the corresponding control air-exposed lungs. The results suggested that NR-DE exposure enhanced 8-OHdG formation in asthmatic lungs. This, at least in part, is involved in the NR-DE-mediated exacerbation of the allergic pathophysiology that was identified in our previous study.

## Introduction

The concentration of particulate matter (PM) with a mass median aerodynamic diameter (a density-dependent unit of measure used to describe the diameter of a particle) ≤2.5 μm (PM_2.5_) is more closely associated with respiratory effects and subsequent mortality than larger particles of mass median aerodynamic diameter ≤10 μm (PM_10_) (1). A noteworthy aspect of the epidemiological data is that health impacts of PM_2.5_ are predominantly identified in subjects with predisposing factors to pneumonia, bronchial asthma, chronic obstructive pulmonary disease, compromised immune disorders and an age of >65 years (2). Diesel exhaust particles (DEPs), the main constituents of PM_2.5_ in urban areas, are epidemiologically considered to be harmful for respiratory systems and diseases (3). In accordance with this, the respiratory toxicity of DEPs has been biologically demonstrated, in the presence or absence of predisposing factors (4–6).

DEP sizes have become progressively smaller due to advancements in the automobile industry, leading to the production and release of particles <100 nm in mass median aerodynamic diameter (defined as nanoparticles). This trend may increase the level of airborne nanoparticles, and consequently possess a greater health concern (7,8). However, there have been few studies that have examined the effects of exposure to relevant types of nano-level DEPs on health, in individuals with or without predisposing factors. We have focused on nanotoxicity in allergic asthma. Since asthma is a chronic airway inflammatory disease and patients with asthma are reportedly highly sensitive to PM (9), we have demonstrated that inhaled nanoparticle-rich DE (NR-DE) exacerbated allergic airway inflammation in mice (10). The aggravation was concomitant with the amplified expression of the allergy-associated cytokines interleukin-5 (IL-5) and eotaxin, in the lung. However, the mechanisms for NR-DE-mediated aggravation of the allergic asthma model have not been fully investigated. The formation of 8-hydroxydeoxyguanosine (8-OHdG) is the main DNA modification induced by reactive oxygen species (ROS) and may be responsible for DNA base mutations. It has been demonstrated that oxidative DNA adducts accumulate and are only repaired through enzyme pathways, resulting in further DNA damage (11). Oxidative DNA damage may be observed in lung inflammation, such as that induced by lipopolysaccharides (12). In addition, 8-OHdG expression is induced or enhanced in the lung as a result of several types of oxidative stress burdens, such as ozone (13), DEPs (14) or asbestos (15), *in vitro* and *in vivo*. In the present study, we investigated the levels of 8-OHdG in the lung by means of enzyme immunoassay (EIA) and immunohistochemistry, to gain insights into the mechanistic pathway of the NR-DE-mediated aggravation of allergic airway inflammation.

## Materials and methods

### Animals

Female Institute for Cancer Research (ICR) mice (age, 6 weeks; weight, 29–33 g; Clea Japan, Inc., Tokyo, Japan) were used in this study. The mice were housed in an animal facility maintained at 24–26°C with 55–75% humidity and a 12 h light/dark cycle, and fed a commercial diet (Clea Japan, Inc.) with *ad libitum* access to water.

### Generation of NR-DE inhalation systems

An 8-l diesel engine (J08C; Hino Motors, Ltd., Hino, Japan) was used for generating the nanoparticles as previously described (10,16). Four exposure chambers were set according to the conditions of the gases, and included a control (control air: CA), low-concentration (36.3 μg/m^3^: D1) NR-DE, high-concentration (168.8 μg/m^3^: D2) NR-DE and high concentration (168.8 μg/m^3^: D3) NR-DE without particulate components. In each inhalation chamber, the temperature and relative humidity were maintained at 20°C and 50%, respectively.

### Study protocol

The mice were exposed to one of the four different gas compositions (CA, D1, D2 and D3) in each chamber system for 5 h/day, 5 days a week for 8 weeks. During inhalation exposure, 1 μg/body of ovalbumin (OVA) or vehicle [phosphate-buffered saline (PBS)] was intratracheally administered every 2 weeks (a total of five times). Finally, the mice were divided into eight groups, sacrificed and studied 24 h following the final intratracheal instillation (80 mice in total). The animal studies were approved by the Institutional Review Board of the National Institute for Environmental Studies, Tsukuba, Japan.

### Bronchoalveolar lavage (BAL) procedure and 8-OHdG level in the BAL fluid (BALF)

The mice were sacrificed by etherization and exsanguination from the abdominal aorta 24 h following the final intratracheal administration. A cannula was inserted into the trachea and secured with a suture. The lungs were lavaged three times with 1.2 ml sterile saline at 37°C, which was instilled bilaterally with a syringe. The fluid was harvested by gentle aspiration. The collected fluid was cooled and centrifuged at 300 × g for 10 min, as described previously (17–19). The collected supernatants were used for an EIA study (n=6 in each group). In another experiment, the lungs were removed for immunohistological examination (n=4 in each group).

The EIA for determining the 8-OHdG level in the BALF was conducted on the basis of the competition between 8-OHdG and an 8-OHdG acetylcholine esterase conjugate (the 8-OHdG tracer) for a limited concentration of 8-OHdG monoclonal antibody. As the concentration of 8-OHdG varies, the concentration of the tracer that is able to bind to the 8-OHdG monoclonal antibody is inversely proportional to the 8-OHdG level. Following incubation with the tracer, antibody and standard or sample in 96-well plates, the plates were washed to remove any unbound reagents, prior to Ellman’s Reagent being added to the well. Finally, the product of this enzymatic reaction was read at 412 nm with conversion to pg/ml, using values obtained from the standard with limits of detection of 30 pg/ml.

### Immunohistochemistry

The degree of expression of 8-OHdG and its localization in the lungs were detected by immunohistochemistry using anti-8-OHdG monoclonal antibody (N45.1, Japan Institute for the Control of Aging, Nikken SEIL Co., Ltd., Fukuroi, Japan; n=4 in each group). The excised mouse lungs were embedded with paraffin. Following deparaffinization, the tissue sections were incubated with anti-8-OHdG antibody (dilution, 1:100) overnight at 4°C, then reacted with biotinylated secondary anti-mouse IgG antibody (Vectastain Elite ABC kit; Vector Laboratories, Inc., Burlington, Canada) for 30 min at room temperature. Streptavidin was added and the color was developed with 3,3′-diaminobenzidine (DAB). Subsequently, the tissue sections were counterstained with hematoxylin (Merck, KGaA, Darmstadt, Germany) and examined by two researchers independently.

### Statistical analysis

Data are presented as the mean ± standard error. Differences between groups were determined using analysis of variance (the Student’s t-test). P<0.05 was considered to indicate a statistically significant difference.

## Results

**Figure 1:**
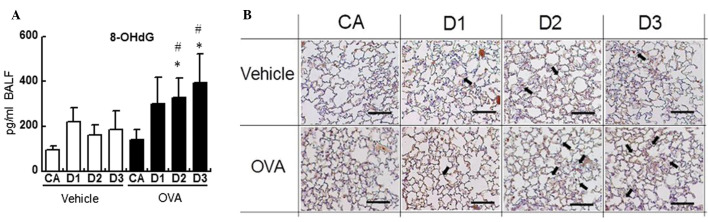
(A) Effects of inhalation exposure to nanoparticle-rich diesel exhaust (NR-DE) on 8-hydroxydeoxyguanosine (8-OHdG) levels in the bronchoalveolar lavage fluid (BALF). Institute for Cancer Research (ICR) mice were exposed to four patterns of gases [control air (CA); low-concentration diesel exhaust (DE; D1); high-concentration DE (D2); and high-concentration DE without particulate components (D3)] over a period of 8 weeks, and were simultaneously intratracheally administered vehicle or ovalbumin (OVA). BAL was performed 24 h following the final intratracheal administration, and the 8-OHdG in the BALF was analyzed using an 8-OHdG assay kit. Results are presented as the mean ± SE (n=6 in each group). ^*^P<0.05 vs. the corresponding vehicle group and ^#^P<0.05 vs. CA-OVA. (B) Effects of inhalation exposure to NR-DE on 8-OHdG formation in the lung. Lungs were removed 24 h following the final intratracheal administration, then fixed and immunohistochemically stained using an anti-8-OHdG polyclonal antibody (n=4 in each group). Representative photomicrographs of the lung sections are shown. Arrows denote positive staining. Scale bar, 100 μm (original magnification, ×100).

We first quantified the level of 8-OHdG in the BALF. The level of 8-OHdG was higher in the D1- (299 pg/ml BALF), D2- (375 pg/ml BALF; P<0.05) and D3- (394 pg/ml BALF; P<0.05) OVA groups than in the corresponding vehicle groups (220, 160 and 186 pg/ml BALF, respectively), and was significantly higher in the D2- and D3-OVA groups than in the CA-OVA group (140 pg/ml BALF; P<0.05; Fig. 1A).

Subsequently, we investigated the expression levels and localization of 8-OHdG in the lung specimens by means of immunohistochemistry. NR-DE plus OVA exposure induced moderate staining for 8-OHdG, compared with that of NR-DE alone or CA plus OVA exposure (Fig. 1B). The 8-OHdG expression was mainly localized to inflammatory polymorphonuclear leukocytes, such as neutrophils and eosinophils.

## Discussion

Although DEPs >200 nm in size have been demonstrated to induce adverse effects on several respiratory diseases, there have been few studies concerning the effects of DEPs containing mainly nanoparticles on lung pathology. Our previous study revealed that inhaled NR-DE exacerbated allergic airway inflammation in mice (10). The aggravation was concomitant with the enhanced expression of allergy-associated cytokines, interleukin-5 (IL-5) and eotaxin, in the lung. However, the mechanisms of the NR-DE-mediated aggravation of allergic asthma have not been fully investigated.

Oxidative stress, such as that due to ROS, is considered to be important in the pathogenesis of various types of lung inflammatory diseases, including allergic asthma (20,21). In addition, a possible association between PM_2.5_ and oxidative stress has been identified. For example, environmentally relevant concentrations of PM_2.5_ have been demonstrated to exacerbate the airway inflammatory response with an increased generation of free radicals in asthmatic patients (22,23). On the other hand, ROS may cause oxidative DNA modification, such as 8-OHdG formation (24). 8-OHdG is also induced by several types of oxidative stress-producing pollutants, such as ozone (13), DEPs (14) or asbestos (15), *in vitro* and *in vivo*. Concordant with these findings, it has been demonstrated that certain types of nanoparticles (carbon black nanoparticles and single/multi-wall nanotubes) were capable of increasing the expression of 8-OHdG in the lung, in association with aggravated lung inflammation or injury (19,25,26). In the present study, we quantified the level of 8-OHdG in the BALF. The level was greater in the D1-, D2- and D3-OVA groups than in the corresponding vehicle groups, and was significantly greater in the D2- and D3-OVA groups than in the CA-OVA group (P<0.05). Furthermore, the immunohistochemical analysis revealed that NR-DE plus OVA exposure induced moderate staining for 8-OHdG, compared with that of NR-DE alone or CA plus OVA exposure. However, no differences in staining were observed among the NR-DE plus OVA groups. These results suggested that NR-DE exposure increased 8-OHdG synthesis and release in the lung, which, at least in part, was involved in the NR-DE-mediated aggravation of the allergic pathophysiology that was identified in our previous study (10). The significant difference between the two parameters (EIA versus immunohistochemical staining) may be due to the time lag during translocation from the lung parenchyma to the bronchoalveolar spaces. Therefore, time-course studies may be required to increase understanding of the process.

Notably, gaseous components in the high-concentration NR-DE without particulate components (D3) significantly elevated the 8-OHdG levels in the BALF in the presence of allergen compared with CA (P<0.05). This is concordant with the allergic pathophysiology observed in the preceding study (10). 8-OHdG synthesis in the lung has been demonstrated to be induced or amplified as a consequence of DNA damage following exposure to gaseous pollutants, such as nitrogen oxides (NOx), sulphur oxides (SOx) or ozone (23,27). Therefore, these gaseous components of NR-DE may be responsible for the enhanced formation of 8-OHdG. However, in the present study, particulate matter, NR-DEP, collected in the inhalation systems, independently elevated the 8-OHdG levels in the presence of OVA (data not shown), as we have previously identified in a study concerning nanoparticles (19). These results suggest that the mechanism of 8-OHdG-hyperproduction may differ between high-concentration DE with and without particulate components.

NR-DE exposure significantly elevated the 8-OHdG level in the lung in the presence of an allergen (as compared with CA exposure). These results suggested that amplified 8-OHdG formation in asthmatic lungs, at least in part, is involved in the NR-DE-mediated aggravation of the allergic pathophysiology observed in our preceding study (10).

## Abbreviations

NR-DE: nanoparticle-rich diesel exhaust
8-OHdG: 8-hydroxydeoxyguanosine
ROS: reactive oxygen species
PM: particulate matter
DEP: diesel exhaust particles
PM_2.5_: PM with a diameter <2.5 μm
NOx: nitric oxides
SOx: sulphur oxides
PBS: phosphate-buffered saline
OVA: ovalbumin
BAL: bronchoalveolar lavage
BALF: BAL fluid
IL: interleukin

## References

[1] Peters A, Wichmann HE, Tuch T, Heinrich J, Heyder J (1997). Respiratory effects are associated with the number of ultrafine particles. Am J Respir Crit Care Med.

[2] Dockery DW, Pope CA, Xu X (1993). An association between air pollution and mortality in six U.S. cities. N Engl J Med.

[3] Patel MM, Chillrud SN, Correa JC (2010). Traffic-related particulate matter and acute respiratory symptoms among New York City area adolescents. Environ Health Perspect.

[4] Ichinose T, Furuyama A, Sagai M (1995). Biological effects of diesel exhaust particles (DEP). II Acute toxicity of DEP introduced into lung by intratracheal instillation. Toxicology.

[5] Takano H, Yoshikawa T, Ichinose T (1997). Diesel exhaust particles enhance antigen-induced airway inflammation and local cytokine expression in mice. Am J Respir Crit Care Med.

[6] Maejima K, Tamura K, Nakajima T (2001). Effects of the inhalation of diesel exhaust, Kanto loam dust, or diesel exhaust without particles on immune responses in mice exposed to Japanese cedar (*Cryptomeria japonica*) pollen. Inhal Toxicol.

[7] Timonen KL, Hoek G, Heinrich J (2004). Daily variation in fine and ultrafine particulate air pollution and urinary concentrations of lung Clara cell protein CC161. Occup Environ Med.

[8] Zhu Y, Eiguren-Fernandez A, Hinds WC, Miguel AH (2007). In-cabin commuter exposure to ultrafine particles on Los Angeles freeways. Environ Sci Technol.

[9] Kappos AD, Bruckmann P, Eikmann T (2004). Health effects of particles in ambient air. Int J Hyg Environ Health.

[10] Tanaka M, Aoki Y, Takano H (2013). Effects of exposure to nanoparticle-rich or -depleted diesel exhaust on allergic pathophysiology in the murine lung. J Toxicol Sci.

[11] Kuchino Y, Mori F, Kasai H (1987). Misreading of DNA templates containing 8-hydroxydeoxyguanosine at the modified base and at adjacent residues. Nature.

[12] Kawai Y, Morinaga H, Kondo H (2004). Endogenous formation of novel halogenated 2′-deoxycytidine. Hypohalous acid-mediated DNA modification at the site of inflammation. J Biol Chem.

[13] Cheng ML, Ho HY, Huang YW, Lu FJ, Chiu DT (2003). Humic acid induces oxidative DNA damage, growth retardation, and apoptosis in human primary fibroblasts. Exp Biol Med (Maywood).

[14] Sanbongi C, Takano H, Osakabe N (2003). Rosmarinic acid inhibits lung injury induced by diesel exhaust particles. Free Radic Biol Med.

[15] Upadhyay D, Kamp DW (2003). Asbestos-induced pulmonary toxicity: role of DNA damage and apoptosis. Exp Biol Med (Maywood).

[16] Fujitani Y, Hirano S, Kobayashi S (2009). Characterization of dilution conditions for diesel nanoparticle inhalation studies. Inhal Toxicol.

[17] Inoue K, Takano H, Yanagisawa R (2005). Pulmonary exposure to diesel exhaust particles induces airway inflammation and cytokine expression in NC/Nga mice. Arch Toxicol.

[18] Inoue K, Yanagisawa R, Koike K (2010). Effects of carbon black nanoparticles on elastase-induced emphysematous lung injury in mice. Basic Clin Pharmacol Toxicol.

[19] Inoue K, Yanagisawa R, Koike E, Nishikawa M, Takano H (2010). Repeated pulmonary exposure to single-walled carbon nanotubes exacerbates allergic inflammation of the airway: Possible role of oxidative stress. Free Radic Biol Med.

[20] Riedl MA, Nel AE (2008). Importance of oxidative stress in the pathogenesis and treatment of asthma. Curr Opin Allergy Clin Immunol.

[21] Li YJ, Takizawa H, Kawada T (2010). Role of oxidative stresses induced by diesel exhaust particles in airway inflammation, allergy and asthma: their potential as a target of chemoprevention. Inflamm Allergy Drug Targets.

[22] Sierra-Vargas MP, Guzman-Grenfell AM, Blanco-Jimenez S (2009). Airborne particulate matter PM_2.5_ from Mexico City affects the generation of reactive oxygen species by blood neutrophils from asthmatics: an in vitro approach. J Occup Med Toxicol.

[23] Ren C, Fang S, Wright RO, Suh H, Schwartz J (2011). Urinary 8-hydroxy-2′-deoxyguanosine as a biomarker of oxidative DNA damage induced by ambient pollution in the Normative Aging Study. Occup Environ Med.

[24] Kuwano K, Nakashima N, Inoshima I (2003). Oxidative stress in lung epithelial cells from patients with idiopathic interstitial pneumonias. Eur Respir J.

[25] Inoue K, Takano H, Yanagisawa R (2005). Effects of nano particles on antigen-related airway inflammation in mice. Respir Res.

[26] Lai CH, Liou SH, Lin HC (2005). Exposure to traffic exhausts and oxidative DNA damage. Occup Environ Med.

[27] Ren C, Fang S, Wright RO, Suh H, Schwartz J (2011). Urinary 8-hydroxy-2′-deoxyguanosine as a biomarker of oxidative DNA damage induced by ambient pollution in the Normative Aging Study. Occup Environ Med.

